# Trade deregulation and fiscal revenue in selected Pacific Island countries

**DOI:** 10.1371/journal.pone.0315733

**Published:** 2025-01-02

**Authors:** Keshmeer Makun, Baljeet Singh

**Affiliations:** School of Accounting, Finance and Economics, University of the South Pacific, Suva, Fiji; Middle Tennessee State University Jennings A Jones College of Business, UNITED STATES OF AMERICA

## Abstract

This paper examines the revenue implications of trade deregulation in a panel of Pacific Island countries from 2010 to 2021. First, we undertake a cross-country analysis of tax revenue, trade, and tax structure. Secondly, we empirically analyze the effect of trade deregulation on trade tax and overall government tax revenue. We find that as the countries have become more deregulated over the decade, the trade tax revenues and direct income tax revenues have declined whereas domestic indirect tax revenues have increased. The empirical estimation reveals the potential Laffer effect for trade tax revenues with respect to trade openness. The effect of regional trade agreements also shows some support for trade tax revenues. Further, the external public debt has a significant positive effect on aggregate tax revenues, while foreign aid has been significant in explaining the decline in both aggregate tax revenues as well as trade tax revenues in the selected Pacific Island countries.

## 1. Introduction

Trade deregulation, the process of reducing trade barriers, has been a defining feature of global economic policy for several decades. By removing tariffs and trade restrictions, countries seek to enhance efficiency, expansion of trade, and consumer choice and foster economic growth. As shown by the completion of the Uruguay Round in 1994 and the inception of the World Trade Organization (WTO) in 1995, international trade has been one of the common development strategies for many countries. Since then, over 2000 trade and investment treaties have been design and implemented [1]. However, the impact of trade deregulation goes beyond the realm of international trade. Trade deregulation may cause a loss of government revenue as tariffs and taxes are reduced if countries are not able to substitute trade taxes with domestic taxes. As a result, it can have profound consequences for government revenue and fiscal policy. In recent decades many developing countries have experienced substantial internal and external imbalances compounded by rising debt. It is not always possible for these countries to transition to domestic taxes as they are constrained by their structural features to make such adjustments. Besides being economically less developed, these countries are predominantly characterized by the existence of sizable subsistence sectors and relatively large dependence on the export of primary products for revenue. Thus, it is an important consideration for countries with significant fiscal challenges.

Recent studies have examined the effect of trade liberalization in the context of macroeconomic policies on tax revenues. Nashashibi and Bazoni [2] in sub-Saharan African countries, for instance, show that depreciation of exchange rate, worsening terms of trade and import tariff reduction adversely impact tax revenue. Similarly, Khattry and Rao [3] and Cage and Gadenne [4] suggest that trade liberalization can undermine tax revenues. On the other hand, Adam et al. [5] and Gropp et al. [6] conclude that tariff reforms do not lead to declining trade revenue. Agbeyegbe et al. [7] show trade liberalization is not strongly associated with government tax revenue and that the effect of trade reforms depends on how one measures trade liberalization. Rao [8] examining the effect of openness finds that low-income countries face a tradeoff between more liberalized trade and reduced tax revenue. More recently, Dutt and Gallagher [9] concluded that on average trade liberalization has reduced the tariff revenue collection. They also find that trade and investment initiatives are adversely related to fiscal revenue and increase public debt.

This paper aims to examine the effect of trade deregulation on tax revenue in the selected Pacific Island countries (PICs). Trade in the Pacific Island economies is considered to play a critical role in their economic progress. We synonymously use trade deregulation in the context of PICs because many PICs have deregulated their trade but not yet fully liberalized trade. Several studies, notably from the World Bank, have observed features that make Pacific economies unfavourably biased including small domestic market, long market distance, high transport costs, lack of fiscal capacity and vulnerability to external shocks and climate change-related natural disasters [10]. These challenges in the Pacific economies, which are mostly attributed to economic geography, to some extent constrain many countries to effectively participate in international trade. However, the Island countries have remained steadfast in their commitment to regional and global integration to develop their economies [11].

PICs negotiated several bilateral and multilateral trade and investment agreements since gaining independence. For instance, many PICs benefited from 1975 the Lome Convention, which provided preferential access to the European common market for several African, Caribbean and Pacific countries. The Lome convention bolstered Fiji’s Sugar export, while also enhancing the export earnings of coffee, coconut oil and palm oil export in PNG. Solomon Island and Vanuatu saw benefits in their Tuna export. In 1981, several Pacific Island member countries joined the South Pacific Regional Trade and Economic Cooperation Agreement (SPARTECA). SPARTECA provided preference access to the New Zealand and Australian markets, and it particularly benefited Fiji’s Garment industry export. PIC export, particularly Fiji’s garment export benefited from the 1974 Mutti-fiber Arrangement (MFA) which provided Fiji a guaranteed quota for textile access to the US. However, the conclusion of the 1994 Uruguay Round of multilateral trade negotiation and the establishment of WTO in 1995 ended many benefits that PICs enjoyed under preferential trade agreements.

Further, Fiji, Solomon Island and Papua New Guinea joined the General Agreement on Tariffs and Trade (GATT) in 1994 and became a member of WTO in 1996, while Samoa and Vanuatu acceded to GATT and WTO in 2012. Under the specific commitments on Tariff and non-tariff barriers outlined in GATT (1994) and WTO (1995), PICs have substantially reduced maximum tariffs and duties across all categories, causing a significant loss in the tariff revenue. Nevertheless, several PICs have implemented goods and services tax (GST) or value-added tax (VAT) to counter-act the tariff revenue losses. Personal income taxes were also lowered at the same time. Similarly, PICs negotiated several regional and bilateral trade agreements such as the Pacific Islands Countries Trade Agreement (PICTA) and the Melanesian Spearhead Group (MSG) to overcome border costs associated with trade. It is believed that trade integration is essential for Pacific countries to effectively expand their markets and reduce trading and business costs. Kaufmann [12] argues that trade deregulation for Pacific economies is necessary if island countries wish to gain the benefits of integration in the world economy.

Although the effect of trade reforms has been widely analyzed, its impact on fiscal policy stance, particularly in small developing economies has been limited. At least, in the context of PICs, the recent development in trade and investment integration has not been analyzed. There are few reports and studies that have examined the revenue implications of various trade agreements concerning Pacific Island countries, including Scolly et al. [13], Filmer and Lawson [14], and Narsey [15]. However, these studies have examined the potential revenue loss from selected trade agreements in selected countries between certain periods. The findings from these studies provide relatively different results on revenue, leading to inconclusive results. The difference in result could be due to different periods, assumptions and methods used. Further, the findings, being based on simple comparative analysis might present a failure to take into consideration structural characteristics and other economic factors that are likely to be associated with trade reforms and taxes.

This study contributes to the literature in a couple of ways. First, it fills the knowledge gap by undertaking the first formal cross-country and empirical research of tax revenue implications of international trade using selected country-level data in the Pacific context. Second, most of the existing literature only assumes a linear estimation procedure for revenue implication of international trade and ignores the potential non-linear effects. Furthermore, literature on tax performance has often overlooked variables such as aid, exchange rate and public debt, which in small developing countries are important determinants of revenue. Given the Pacific countries are recipients of aid, prone to exchange rate volatility and have experienced elevated debt situations, these variables are examined in the tax effort model. Finally, to examine to extent to which trade deregulation influences tax revenue, the empirical estimation disaggregates trade taxes from total tax revenue. This analysis offers a more comprehensive assessment of the possible revenue effects of trade deregulation, which is helpful for policy design, evaluation, and decision-making.

Foreshadowing some of the main results, the analysis shows that while selected Pacific countries have deregulated their trade over the decades, the trade tax revenue has declined. On the other hand, these countries have witnessed an increase in domestic indirect taxes. Second, structural factors like the level of development have been significant in influencing total tax revenues as well as trade taxes. Third, foreign aid is inversely associated with total tax revenues as well as trade taxes. The external debt stock has a significant positive impact on total tax revenues. Fourth, while a decline in effective trade taxes (openness) is observed to have caused a significant decline in trade tax revenues, the regional trade agreements show some evidence of gaining trade tax revenue. Trade openness is also found to exhibit a nonlinear effect on trade taxes, indicating the presence of a potential Laffer effect. In addition, a stable macroeconomic environment including exchange rate policies has the potential to strengthen trade tax revenue.

The rest of the paper is organized as follows. The next section provides the theoretical and empirical literature on trade reform and tax revenue. Section 3 provides cross country analysis of the changes in tax revenue and tax structure. Section 4 outlines the tax effort model and estimation for empirical analysis. The results of the effect of trade deregulation on tax revenue are discussed in Section 5. Finally, Section 6 concludes the paper with implications.

## 2. Literature review

The theoretical literature on trade deregulation is primarily focused on efficiency gains and potential economic growth benefits. However, trade liberalization is also believed to be associated with tax revenue via international trade taxes [16], although the exact link is conditioned on different factors including features of liberalization, and response of trade (exports and imports) to trade reforms. While the initial step in trade liberalization involves replacing quantitative restrictions with fiscal duties, which could provide high trade taxes, ultimately trade liberalization leads to a reduction of duties, which would impact trade tax revenue [6]. The link between trade liberalization and tax revenue is uncertain and depends on domestic economic development, tax structure and institutional capacities [17]. It is argued that any loss in trade tax due to trade reforms is replaced with national tax policies like value-added tax, known as VAT or other consumption taxes. However, different countries have different taxation policies to collect taxes. Thus, it is not wise to generalize the implication of trade liberalization and other factors on tax revenue in the total.

Aizenman and Jinjarak [18], identified “easy-to-collect” revenues from tariff and seigniorage and “hard-to-collect” taxes from VAT. Their findings based on the hypothesis that globalization potentially leads to a reduction of the countries’ tax revenue, noted that most developing countries shifted their tax to “hard-to-collect”, which resulted in a 9% increase from the hard-to-collect tax sources. In agreement with Aizenman and Jiniarak [18], Baunsgaard and Keen [19] examine whether countries can recover from their loss of trade tax due to liberalization. They examined the full sample as well as separated the countries into income groups (low middle and high). The result noted different degrees of revenue recovery for low and middle income countries. The low-income countries have the lowest recovery rate, while the middle-income countries’ recovery rate is relatively higher.

With the challenges of shifting tax revenue and the longer recovery rate for least developed and developing countries due to trade liberalization, there are a number of empirical studies that have been conducted, particularly on the effect of trade liberalization on tax revenue. Karimi et al. [20], investigate the tax structure of 97 developing countries with the impact of trade liberalization for the period 1993–2012. Their result noted that trade liberalization does not have a significant impact on all kinds of tax shares in developing countries. Rather trade liberalization in the form of reduced tariffs contributed to changing the tax composition of developing countries by moving more towards income and goods and services taxes. The authors noted that trade liberalization in the form of reduced tariffs seems to contribute to enhancing tax structure in these countries. Arezki et al. [21], also shared the same sentiment by noting that trade liberalization may impact the tax structure.

Longoni [22] analysed the relationship between trade liberalization and tax revenue for a panel set of 53 African countries for the period 1970–2000 and noted that there exists a large trade-off between a greater degree of openness to international trade and the revenue collected from export and import taxation. The author’s findings noted the existence of a Laffer effect to which the relationship between trade tax and trade tax revenue is nonlinear. Operating on the rising side of an inverse U-shaped curve indicated that a further decrease in tariff reduction can only lead to a further decrease in trade tax revenue. Therefore, trade openness must be accompanied by a strong reform of the domestic taxation system to avoid increased revenue loss which exacerbates more into precarious fiscal deficit and total debt position for these countries.

Agbeyegbe et al. [7], noted a mixed relationship for trade liberalization, exchange rates and tax revenue for 22 countries in Sub-Saharan Africa. Performing Generalized Method of Moments (GMM) regression to test the relationship, the author finds evidence that the relationship between trade liberalization and tax revenue is sensitive to the measure used but in general trade liberalization is not strongly linked to aggregate tax revenue or its components. Currency appreciation and higher inflation show some linkage to lower tax revenues. [3], examine the argument that trade liberalization depresses tax revenue /GDP ratios in developing countries. Using a fixed-effects regression framework for the period 1970–1998 for 80 developing and industrialised countries, they find that transitioning from trade tax to domestic tax results in falling income and trade tax and the structural characteristics faced in the country have been significant in explaining the decline.

On the other hand, studies such as Greenaway [23]Hitiris, [24] Ram [25]and Tanzi, [26] noted a positive relationship between import, import duties and trade tax revenue. They also found a negative relationship between the level of economic development and trade tax revenue. Similarly, in the context of growing rhetoric against trade, Gnangnon [27] examine the effect of multilateral trade policy reforms on government revenue for 155 countries over the period from 1995–2014. The findings by Gnangnon [27], revealed that multilateral trade liberalization does have a positive impact on government revenue. It is also revealed that the impact appears to be dependent on both the country’s level of development and its domestic trade policy liberalization.

However, in a recent study, Cagé and Gadenne [28] disentangle the different episodes of trade liberalization from the period 1792–2006 and found trade liberalization during pre-1970 was unlikely to impact tax revenues. However, in recent times about 40% of the countries experienced a reduction after the episodes of liberalization. Overall, it was noted that trade liberalization in the 1970s led to a larger decline in trade tax revenue in developing countries than in today’s rich countries. On the impact of the Free Trade Agreement on the Central American Countries, Rodlauer and Schipke [29], noted that despite the different liberalization schedules for the different countries, these countries have a revenue loss due to trade liberalization. This revenue loss ranges from 1.6% of GDP for Nicaragua to 7.5% of GDP for Honduras. Looking at the case of Sub-Saharan Africa, Agbeyegbe, et al. [7], find evidence that the relationship between trade liberalization and tax revenue is sensitive to trade liberalization.

Further, there a numerous studies that have attempted or have designed indicators and measures to evaluate the overall tax performance of a country. In most previous studies, the ratio of tax revenue to GDP has been used as a common index of tax performance or an indicator of a country’s tax effort ([7, 30]). This measure of tax performance (tax ratio) aims to explain the key determinants of differences in tax performance across economies [31]. Several studies have concluded that such differences in tax performances are due to distinct ‘capability’ to tax. For instance, Musgrave [32], one of the earliest studies on this discussed reasons as to why lower-income countries have low tax revenue, whereby he coined the term ‘tax handles’ as a mechanism to increase tax effort. However, for less developed economies it is hard to impose and gather taxes [33]. According to Musgrave [32], it is not only resources and administration in less developed countries that make tax revenue generation difficult but also the various economic structures that offer very few ‘handles’ on which to impose taxes. Hence, the lack of tax handles in these countries could be the reason for low tax ratios.

In the context of the Pacific Island Countries, expected revenue loss due to the reduction or elimination of tariffs has been one of the most frequent issues raised in the discussions of the trading agreements [34]. Besides being heavily dependent on grants and other non-tax revenue, Sy, et al. [35] argue that Pacific Island countries (Fiji, Papua New Guinea, Solomon Islands and Samoa) rely more on tax revenue than on non-tax revenue. Studies examining the empirical relation between trade liberalization and fiscal revenue are sparser. Some reports and studies relying on specific trade agreements at the time of negotiation provide mixed results [13, 15, 34, 36]. Thus, there is no formal consensus and empirical assessment of the effect of trade liberalization on fiscal stance in the Pacific context. This paper builds on the extant literature and examines the interrelationship between trade liberalization and taxes, taking into consideration the structural and other economic factors that can influence the link between international trade and government tax revenue.

## 3. Cross-country analysis of tax revenue and trade deregulation

The five selected Pacific Island countries for which data is available are used in the analysis. The dataset is created using data from the World Bank’s World Development Indicators [37] and the Asian Development Bank’s Key Indicators. In the context of Pacific Island countries, complete and consistent data is one of the key limitations acknowledged by researchers. Even for these selected countries, some data are missing which makes it relatively difficult to trace out consistent trends and characteristics of some of the indicators used. However, with the available information, we make several key and important observations on general trends and characteristics. The objective here is to examine the trade tax characteristics and changes in tax as a result of trade deregulation and taxation structure. The level of trade deregulation or openness is measured by the effective trade tax rate, which is the ratio of trade tax to total trade volume. Thus, a decline in the ratio depicts a greater trade deregulation.

Table 1 shows the inverse relation between trade and trade taxes as revenue sources. While trade volume (TTRADE) has increased over the period, the trade taxes (TRT) in the Pacific Island countries have declined. For instance, in Fiji, about 7% of trade tax (TRT) share in GDP during 1990–1994 declined to around 5% in 2015–2019. A similar trend is observed in the Solomon Islands and Vanuatu. Papua New Guinea has the largest drop in trade taxes (6.67% in 1995–1999 to 0.93% in 2015–2019). The average tariff rates underlying any revenue from trade also show an overall declining trend (see Table A1 in S1 Appendix). This indicates that the rise in trade tax revenue from increased trade volumes is not sufficient to compensate for the loss in trade tax revenue resulting from reduced tariff rates (TLIB), thus causing an overall decline in trade tax revenue as a share of GDP (TOR). The ratio of tax revenue to GDP (TOR) during 2015–2019 stands at around 24% (for Fiji), 23% (for Samoa and the Solomon Islands), 17% (for Vanuatu) and 13% (for Papua New Guinea). Fiji being the upper middle-income country has the highest tax revenue to GDP ratio.

**Table 1:** Trade deregulation and tax structure in selected Pacific Island countries.

Country	Period	TOR	TLIB	TTRADE	IPCTG	IPCTR	DTG	DTR	TRT	TRTTR	IDTG	IDTTR
Fiji (UMI)	1990–1994	21.82	5.72	116.35	8.66	36.63	6.07	28.02	6.69	30.53	12.76	36.73
	1995–1999	na	na	119.55	8.02	36.97	8.01	36.89	na	25.53	13.54	40.71
	2000–2004	na	na	129.21	7.12	31.9	10.61	47.49	na	20.55	15.21	45.70
	2005–2009	na	na	117.21	7.68	34.14	10.51	46.81	na	18.9	14.75	43.26
	2010–2014	22.75	4.10	121.63	6.49	28.59	11.28	49.56	4.97	21.84	16.25	48.65
	2015–2019	23.71	5.13	101.06	6.71	28.25	11.82	49.9	5.17	21.84	17	48.03
	2020–2021	17.58	5.28	76.81	5.12	29.31	8.47	48.22	3.99	22.46	12.46	53.11
Samoa (LMI)	2000–2004	na	na	78.91	na	na	na	na	na	na	na	na
	2005–2009	na	na	78.35	na	na	na	na	na	na	na	na
	2010–2014	22.31	3.03	79.09	5.42	24.34	14.33	64.25	2.42	10.81	16.74	52.75
	2015–2019	23.44	3.29	77.22	5.28	22.54	15.51	66.16	2.53	10.82	18.05	53.54
	2020–2021	24.72	3.79	69.18	5.74	23.21	16.42	66.44	2.56	10.35	18.98	52.07
Solomon Islands (LMI)	2005–2009	na	na	75.87	na	na	na	na	na	na	na	na
	2010–2014	25.88	10.27	100.25	8.85	34.54	6.39	24.85	10.37	40.05	16.79	39.01
	2015–2019	24.42	11.43	86.25	8.34	34.01	5.91	24.17	9.86	40.39	15.77	40.14
	2020–2021	20.86	13.46	64.79	7.28	34.57	4.79	22.79	8.64	41.05	13.43	42.78
Vanuatu (LMI)	1990–1994	na	na	97.85	na	na	6.34	31.39	na	68.6	20.19	79.81
	1995–1999	17.88	10.22	93.41	na	na	8.07	45.29	9.56	53.3	17.63	80.71
	2000–2004	na	na	91.38	na	na	na	na	na	na	na	na
	2005–2009	16.49	5.67	98.83	na	na	10.29	62.41	6.15	37.34	16.45	83.25
	2010–2014	17.32	3.65	103.41	na	na	13.17	76.02	3.77	21.87	16.95	80.57
	2015–2019	17.25	3.47	105.17	na	na	13.09	75.85	3.64	21.12	16.73	79.72
	2020–2021	14.04	2.38	61.72	na	na	10.57	75.32	2.99	21.31	13.56	82.59
Papua New Guinea (LMI)	1990–1994	19.31	6.51	92.08	10.11	52.34	2.67	13.86	5.99	31.01	8.66	25.56
	1995–1999	21.67	6.32	106.12	12.15	56.07	2.19	10.19	6.68	30.92	8.88	19.46
	2000–2004	21.56	5.29	121.29	11.76	53.92	2.95	13.71	6.21	29.35	9.15	21.51
	2005–2009	na	na	na	na	na	na	na	na	na	na	na
	2010–2014	17.91	2.00	53.61	11.86	66.25	5.04	28.18	0.97	5.42	6.02	15.69
	2015–2019	13.39	1.67	55.45	8.04	59.86	4.41	33.08	0.93	6.94	5.34	26.61
	2020–2021	11.88	1.64	51.92	6.86	57.83	4.08	34.41	0.92	7.76	5.01	30.36

Source: authors calculations. Note: UMI- upper middle income. LMI–lower middle income. TOR—total government tax revenue % GDP, TLIB–international trade taxes % total trade, TTRADE–total trade as % of GDP, IPCTG–income, profits and capital gains tax (GDP %), IPCTR—income profits and capital gains tax (% tax revenue), DTG–domestic tax on goods and services (% GDP), DTR—domestic tax on goods and services (% total tax revenue), TRT- international trade tax % GDP, TRTTR–international trade tax as % total tax revenue, IDTG–indirect tax as % GDP (DTG+TRT), IDTTR—indirect tax as % total tax revenue (DTR+TRT-TOR). International trade tax includes import duties, export duties, exchange profits, and exchange taxes. Domestic taxes on goods and services include general sales and turnover or value-added taxes, selective excises on goods, selective taxes on services, taxes on the use of goods or property, taxes on extraction and production of minerals, and profits of fiscal monopolies (World Development Indicators, 2023).

Pacific Island countries also exhibit different economic structural features. Some are less urbanized, have different population sizes and dependent on high-income countries like Australia, New Zealand and the US for their exports, and aid, than others. As expected, trade deregulation has a bearing on fiscal outcomes. As Pacific Island countries become increasingly open to international markets, the ratio of tax revenue to GDP (TOR) has declined. For instance, lower-middle-income countries like Solomon Islands Vanuatu and Papua New Guinea experienced approximately 5.6%, 0.4%, and 25% decline in their tax revenue to GDP ratio, respectively during the period 2010–2014 to 2015–2019. This was not the case for Fiji and Samoa during the same period as their openness to trade (measured by effective trade tax rate- TLIB)) increased during that period. However, taking the whole period (1990–2021), it is observed that increasing openness has resulted in declining tax revenue (TOR) in upper-middle-income countries like Fiji. Certainly, other factors than structural features may have contributed to this. The decline in revenue has implications for public spending. However, the public expenditure has increased despite international financial agencies’ recommendations to rein in fiscal deficits. As a result, indebtedness in the Pacific Island countries has increased substantially [38, 39].

Table 1 also indicates the tax structure in the selected Pacific Island countries. As shown in the table, the share of income taxes (IPCTG) as a percent of GDP has declined with level development. It is observed that in Fiji and Samoa, direct taxes (income) contribute far less than indirect taxes. For instance, during 2015–2019, the IPTCG was 6.71% and DTG was 11.82% (for Fiji), IPTCG was 5.28% and DTG was 15.51% (for Samoa) whereas the IPTCTR was 28.25% and DTR was 49.9% (for Fiji) and IPTCTR was 22.54% and DTR was 66.16% (for Samoa). This suggests that even though the income tax rates have been lowered, the taxable income has not seen substantial growth, potentially reflecting the country’s inability to raise income tax and the possibility of tax evasion. In the 2023 Fiscal Review Report found that only 22000 individuals pay income tax in Fiji. During the same period in the Solomon Islands and Papua New Guinea, income (direct) tax contributed more than the indirect taxes although the trend is declining. However, the general trend is, that as per capita income increases, indirect taxes contribute gradually more as a share of tax revenue in the Pacific Island countries.

The difference between the countries regarding trade taxes is also borne in Table 2. While in some countries trade taxes contribute around 40% of tax revenue, this statistic is less than 10% in some other countries. However, domestic taxes (DTG and DTR) do not show such a relation. Although some less developed Pacific Island countries have a very low share of indirect taxes in GDP (5.91% -Solomon Island, 4.41% -Papua New Guinea) and relatively developed countries have higher shares (15.51%—Samoa. 13.09%—Vanuatu, 11.82%—Fiji) during the 2015–2019, the relative share of domestic indirect taxes in total tax revenue ranges from approximately a quarter to three quarters in Pacific countries. Compared to other countries, Samoa and Vanuatu are observed to raise more revenues from DTR (domestic indirect taxes) while indirect tax as a ratio of GDP is relatively similar to other countries. While indirect tax collection is more important in overall tax revenue in Pacific Island countries, direct taxes remain equally vital in lower-income Pacific countries like the Solomon Islands and Papua New Guinea. Moreover, it is also found that in almost all Pacific countries as the trade taxes share in GDP (TRT) declines, the domestic indirect taxes share in GDP increases.

**Table 2:** Taxation and trade openness (% change between 1990–94 and 2015–19).

Country	TOR	IPCTG	DTG	TRT	IDTG
Fiji	8.66	-22.52	94.73	-22.72	33.22
Samoa	5.06*	-2.58*	8.23*	4.55*	7.83*
Solomon Islands	-5.64*	-5.76*	-7.51*	-4.92*	-6.07*
Vanuatu	-3.52	na	106.5	-61.92	-17.14
Papua New Guinea	-30.65	-20.47	65.17	-84.47	-38.34

Source: Author’s calculations.

* is the % change between 2010–14 and 2015–19.

Table 2 provides the summary of changes in tax structure between 1990–94 and 2015–19. Some main observations are as follows. A decline in total tax revenue in the share of GDP has taken place not only because of falling trade taxes (TRT) in most countries but also as a consequence of a fall in direct income taxes. This outcome in tax change is not only transpired by changes in market outcome where tax evasion can be prevalent and lack of high paying jobs but also structural features of the countries that limit states to tax more income. Further, while trade tax has declined, domestic goods and services tax increased in Pacific Island countries. This is possible due to the introduction and gradual increase in these taxes over time and increased consumption of imported goods due to the removal of trade barriers.

Even though the revenue (TOR) for Fiji has increased, the share of direct tax (IPCTG) in GDP has declined by 22.52%, while trade income (TRT) as a share of GDP declined by 22.72%. Taxes on goods and services (DTG) as the ratio of GDP has increased remarkably. Overall, the net 33.22% increase in indirect taxes has caused the observed growth in total tax revenue (TOR) of 8.66% between 1990–94 and 2015–19.

The tax revenue in Vanuatu and Papua New Guinea has declined mostly due to negative changes in trade and direct income taxes. While income and trade taxes decline, the domestic tax (DTG) has increased, however, it is not sufficient to offset the fall in trade and income taxes. As a result, indirect tax as a percentage of GDP (IDTG) in these two countries experienced an overall decline during 1990–94 and 2015–19.

## 4. Empirical analysis

### 4.1. Model and estimation

Based on the literature and cross-country analysis of the trade tax revenue and trade, we empirically examine various propositions of tax revenue and trade relationship. Several studies have used a variety of methods to estimate tax effort. The conventional approach is regressing the tax performance (ratio) on factors that constitute a country’s tax handles. In other words, the independent regressors employed are the determinants of the tax revenue. Based on this conceptual framework, the following is the postulation of the tax effort function:

$$\frac{TR}{Y}=f(Z_{i},\ldots .Z_{s},v)$$ (1)

Where TR is the country’s tax revenue, Y is the level of income (measured by GDP) and TR/Y is the tax ratio. *Z*_*i*_,..*s* represents the independent regressors expected to affect the TR/Y. *v* is the unobserved error variable. The common independent or explanatory variables in the tax effort model are based on the country’s economic structure and other macroeconomic factors. These variables are considered to represent the idea that tax handles have implications for the level of tax revenue generated. Previous works have widely used the following structural and macroeconomic factors in the tax model. i) Economic development, represented by per capita GDP. High-income levels reflect higher economic development. Consequently, there is a high capacity to generate and pay tax [3, 6]. Further, developed countries have enhanced tax administration and progressive tax systems [7]. Therefore, a positive relation is conjectured between tax revenue and per capita income. ii) Openness or trade deregulation. For fiscal performance and external sector studies, openness has been considered as one of the important factors in analyzing growth and taxation [40, 41]. However, one of the key issues is the formulation of an appropriate measure for openness. Numerous previous studies have constructed and used various measures depending on the purpose of the research. The relevance of these proxies depends on various factors and is explained in the IMF [42]. Adam et al.[5]and Gropp et al. [6] use the traditional measure defined as the export and import share of GDP. In addition to this, Gropp et al. [6] used tariff rates as a share of import duties in value imports. More recently, Khattry and Rao [3] in analyzing tax performance used the ratio of trade taxes to trade volumes (effective tax rate) to measure openness. A decline in the trade tax ratio indicates greater openness. Given that small developing countries with less domestic tax handles have a high dependency on the external sector, an increase in openness is likely to influence their tax revenue [18].

In this study, we extend the tax effort model to investigate the impact of trade deregulation tax revenues in the selected Pacific Island countries. In addition to the variables mentioned above, additional indices are introduced. Further, to examine the effects of trade deregulation on different tax components, this study disaggregates trade taxes from total tax revenue.

Following on from the previous discussion, since economic development is positively related to tax revenue, it may not be the case with trade tax. This is perhaps because developed countries might have relatively less reliance on trade taxes [18]. Further, the relationship between openness (measured by effective taxation rate) and trade tax can potentially exhibit a nonlinear relationship, as an excessive rise in trade tax rates could lead to a reduction in trade revenue [3, 6]. Thus, to consider possible nonlinearity, a quadratic version of trade deregulation is used to measure its effect on trade tax income. Mathematically, the revenue maximization function of trade deregulation (openness) is derived as follows:

$\gamma_{i}+{2\gamma }_{sq}(open)=0$, solving for *open* gives the following expression: $open=\frac{{-\gamma }_{i}}{{2\gamma }_{sq}}$

Where *γ* is the estimate parameter and *open* is the measure of trade deregulation.

Further, previous literature on tax performance has often overlooked some important variables such as aid, exchange rate and public debt, which in developing countries could be important determinants of revenue generation. Given the Pacific countries are recipients of aid, prone to exchange rate volatility and have experienced elevated debt situations, these variables are included in the extended model. Thus, based on these premises and related literature, the following extended tax effort model is examined for the Pacific Island countries:

$${TREV}_{it}=\gamma_{0}+\gamma_{1}{GDPC}_{it}+\gamma_{2}{AID}_{it}+\gamma_{3}{EXD}_{it}+\gamma_{4}{OPEN}_{it}+\varepsilon_{it}$$ (2)

$${TTAX}_{it}=\gamma_{0}+\gamma_{1}{GDPC}_{it}+\gamma_{2}{AID}_{it}+\gamma_{3}{EXD}_{it}+\gamma_{4}{OPEN}_{it}+\gamma_{5}{OPEN}_{it}^{2}+\varepsilon_{it}$$ (3)

$${TTAX}_{it}=\gamma_{0}+\gamma_{1}{GDPC}_{it}+\gamma_{2}{AID}_{it}+\gamma_{3}{EXD}_{it}+\gamma_{4}{ER}_{it}+\gamma_{5}{TA}_{it}+\varepsilon_{it}$$ (4)

Where *TREV* is total tax revenue as a share of GDP and *TTAX* is the trade tax revenue as a ratio of GDP. GDPC is the level of economic development and is measured by per capita income. *AID* represents official development assistance received by countries. Aid and grant assistance have been an important source of financing for public expenditure in the Pacific Islands for decades [43]. Countries’ dependency on aid can potentially weaken state institutions, and government accountability, raise rent-seeking behaviour, and reduce the effort and incentive to undertake reforms of existing policies, leading to a reduction in tax revenue collection [44, 45]. Further, aid could also reduce governments’ dependence on domestic taxes or even be a conduit to reduce domestic tax burden [46]. Thus, a negative effect of aid is expected on tax revenue. EXD is the external debt stock as a percent of GDP. In a lot of countries, particularly in less developed countries, a large demand for public expenditure results in fiscal deficits and as such a high public debt occurs [47]. To service debts, current tax income can be used to pay both interest and principal. However, with high public debt obligations, it may prompt the government to increase taxes. Hence, it is presumed that debt is positively related to tax revenue collection. For the trade tax model, the foreign exchange rate plays a key role in determining the tax receipt. Given trade tax is dependent on the level of tariff, which relies on the import volume, any variation in the exchange rate could impact the level of imports and consequently trade taxes. ER is the exchange rate represented by the average national currency per US dollar. Further, since trade deregulation takes place in many different forms, the analysis of trade tax is also examined with additional measures of trade deregulation. The recent practice of trade openness, preferential trade agreement (TA) or regional trade agreements as they are called in the Pacific, is introduced and examined in the model. As countries deregulate their trade in terms of access to overseas markets and concessional commodity prices, trade can help enhance revenue. In countries where the fiscal imbalance is huge and trade taxes are a significant source of revenue, trade agreements could have a substantial impact on aggregate revenue [6].

### 4.2. Data properties and cointegration

The study is based on five selected Pacific Island countries (Fiji, Samoa, Solomon Islands, Vanuatu, and Papua New Guinea) with available data for the period 2010–2021. All data used in the study are obtained from the World Bank’s World Development Indicators, except for the trade agreement, which is sourced from the World Trade Organization database. The variables are used in the natural logarithm form. A dummy variable for TA is used where a value of one is observed for a trade agreement that is signed and in force and zero otherwise.

The empirical analysis starts with the investigation of the unit root properties of the variables used in the study. Any instability in the time series can lead to pseudo regression issues. In the context of the panel study, unit root test design by Levin et al. [48] and Im et al. [49] is utilized for this purpose. Table A2 in S1 Appendix provides the results of the Levin et al. [48] and Im et al. [49] panel unit root tests. The results show mixed outcomes in terms of the stability properties of the series. However, most of the statistics show that variables follow the I (1) process in difference order at a given significance level. This finding implicates possible long-run cointegration between the variables.

Next, Pedroni’s [50] cointegration method is utilized to investigate the presence of long-run equilibrium relationships among the variables in tax effort models. This method offers seven test statistics that allow for heterogeneity. It also considers fixed effects while including trends and allowing long run estimates to vary. Table A3 in S1 Appendix provides the results of Pedroni and Kao cointegration tests. For the Pedroni test, seven different statistics are calculated from residual resulting from cointegration regression. Of these seven statistics, four are from in-group (panel-v, panel-rho, panel-pp and panel-ADF) while others are from intergroup (Rho, group-pp and group-ADF). Based on these results, four out of seven test statistics are significant, rejecting the null hypothesis and supporting the cointegration relationship between the variables in the three models. Similarly, the null hypothesis in the Kao test is rejected, suggesting regression residuals are stable and the presence of long run relationship between variables.

## 5. Main result and discussion

Following this, the effect of trade deregulation on tax revenue is estimated. As indicated above, this paper estimates two main models, drawing from the conceptual framework. In the first model (Equ (2)), aggregate tax revenue is modelled as a function of trade deregulation and other variables while the second model (Eq (3)), focuses on the implication of trade deregulation on trade tax revenue. The second model also accounts for the potential asymmetric effect of trade openness on taxes. The third model (Eq (4)) considers the impact of exchange rates and recent developments in trade agreements. The estimation includes the fixed effect regression, which allows for unobservable time and country-specific factors [3]. Further, Hausman test for preferred estimator favored the fixed effect, thus random effect results are not reported. However, the possible bias from serial correlation in error residual (*ε*_*it*_) and potential endogeneity of trade deregulation variables can pose problems for fixed effect estimators. While the first of these two issues may not be too challenging since it can be addressed by auxiliary regression and checking the first-order serial correlation [51], the presence of potential endogeneity could be problematic. To address this, along with fixed effect results, additional estimator-two-stage least squares (2SLS) results are reported for the tax effort models. For Eqs (3) and (4) the lagged values of trade (Open) and GDP per capita (GDPC) variables are used as instrument, respectively and tested by Sargan test.

Table 3 provides the results of the panel fixed effect and two-stage least square estimates for the tax effort models. For Eq (2) where the dependent variable is total tax revenue as a share of GDP (TREV), the coefficient estimates are as anticipated, broadly. The tax revenue increases with the level of economic development (GDPC). The significant positive effect on total tax revenue of GDPC indicates the country’s ability to generate and collect more tax revenue as the income increases. Given the majority of the countries in the sample are from low-income countries, this finding is consistent with prior studies [3, 6] which show that in low-income countries, rising income led to a rising share of tax revenue/GDP. As anticipated, foreign aid (AID) tends to adversely impact tax revenue in these countries. The negative and statistically significant effect of aid suggests that these countries do depend on foreign aid as a source of government revenue in addition to domestic taxes. The effect of external debt (EXD) on tax revenue is positive, implying an increase in debt stock leads to an increase in taxes. This assessment provides support that to meet debt obligations, small developing countries usually raise revenue from taxes. With respect to trade deregulation (Open), which is the key variable in the analysis, both the estimate shows a positive coefficient-implying trade deregulation has led to declining tax revenue. The result indicates that a 1% increase in openness (reduction in trade taxes) contributes to about a 0.06% reduction in tax revenue. Given the low tax base of this country group, the findings reflect these countries can lose a substantial amount of tax revenue by way of lowering trade taxes. For instance, the average trade tax accounts for about 22%, 11%, 40%, 21%, and 7% for Fiji, Samoa, Solomon Islands, Vanuatu, and Papua New Guinea, respectively in total tax revenue, during 2015–2019.

**Table 3:** Effect of trade deregulation on tax revenue.

	Eq 2 (LTREV)		Eq 3 (LTTAX)		Eq 4 (LTTAX)	
	Panel Fixed	2SLS	Panel Fixed	2SLS	Panel Fixed	2SLS
*LGDPC*	0.196 (0.009)**	0.298 (0.000)*	0.067 (0.692)	0.341 (0.071)***	0.369 (0.044)**	0.734 (0.065)****
*LAID*	-0.097 (0.011)**	-0.206 (0.000)*	-0.094 (0.037)**	-0.049 (0.018)**	-0.097 (0.052)**	-0.142 (0.021)**
*LEXD*	0.126 (0.093)***	0.221 (0.006)**	0.029 (0.803)	0.206 (0.546)	0.128 (0.248)	-0.101 (0.734)
*LOpen*	0.043 (0.000)*	0.055 (0.000)*	0.049 (0.020)**	0.179 (0.084)***		
*Open* ^*sqd*^			-0.0018 (0.085)***	-0.006 (0.066)***		
*LER*					-0.093 (0.004)**	-0.110 (0.037)**
*TA*					0.101 (0.046)**	0.048 (0.133)
*Constant*	1.689 (0.004)**	1.739 (0.001)**	1.745 (0.009)**	1.343 (0.836)	3.329 (0.143)	3.077 (0.536)**
*R* ^*sqd*^	0.691	0.658	0.726	0.794	0.879	0.886
Serial correlation (p-value)	0.117		0.188		0.108	
Sargan Test (p-value)		0.866		0.924		0.468
No. of observation	50	50	50	50	50	50
No. of countries	5	5	5	5	5	5

Note: Probability values are in parentheses and significance level is indicated by

* (1%), ** (5%) and *** (10%).

Eq (3) examines the effect of trade deregulation on trade taxes. Interestingly, the structural effect continues to exert a relatively strong impact on the trade tax as well. There is a positive relationship between income and trade tax revenue. This possibly reflects the fact that given low-income levels in these countries, an increase in income would facilitate more trade and hence more trade taxes. This finding also suggests that although dependency on trade taxes has reduced (as indicated in Table 1), this country group could gain tax revenue from trade through income channels. Similar to Eq (2), foreign aid is negatively associated with trade tax revenue. However, compared to the total tax revenue, foreign aid has a small magnitude effect on trade taxes. Although external debt is statistically insignificant, the estimated sign suggests trade tax increases as countries’ debt stock increases. Openness, which measures trade deregulation has a similar negative effect on trade taxes as well. This estimated outcome is indicative of a possible tradeoff between trade deregulation (reduced protection) and reduced trade tax revenue. Nashashibi and Bazoni [2] and Cage and Gadenne [28] also find inverse relation between trade liberalization and tax revenue. It is also observed that the effect of openness appears to be more pronounced in trade tax. *Open*^*sqd*^, which captures the potential nonlinear effect of trade deregulation and a negative and statistically significant magnitude. This finding supports the possible existence ‘Laffer effect’ for trade taxes. The revenue maximization or the optimum rate for trade tax is estimated to be 7%. However, as depicted in Table 1, the rate of trade taxation is below this estimated rate in all countries except for the Solomon Islands during 2010–2021. Thus, it is clear that most of these countries have been taxing below the optimum rate.

Further, Eq (4) examines the effect of exchange rates and trade agreements on trade tax revenue. The effect of the exchange rate is negative and statistically significant in both estimates, indicating appreciation of currency would increase import volume, and thus contribute to the trade tax revenue. The effect of trade agreement is found to be positive on trade tax in the case of this country group, as expected, however, it is statistically significant in only one of the estimates. While most trade agreements seek to remove trade barriers like trade tariffs, this finding possibly points to the fact that this country group being a small player in the world market with low terms of trade is in the process of fully developing its trade sector and likely to benefit more from creating and expanding volumes of trade. Further, most of the agreements are preferential arrangements that allow many island nations some free market access to developed countries.

## 6. Conclusion and implications

Over the past number of decades, most countries have experienced a rapid pace of integration into the global economy through trade deregulation, which has provided numerous benefits to these countries. The rapid increase in globalization has shown a considerable increase in international trade, and output, and subsequently, an increase in economic welfare. While trade has reduced the constraints to progress (through access to capital, technology, input import and access to markets), it is essential to ensure that trade deregulation does not worsen the fiscal constraints, especially in small developing countries that are challenged by limited tax handles and growing debt.

This paper has examined the implication of trade deregulation on government revenue in selected Pacific Island countries. The study aims to bridge the gap in the extant literature, which has not explored this subject in the Pacific context. The analysis is based on a sample of five selected countries on which data is available. The cross-country analysis shows that the trade tax revenue has declined and domestic tax revenue has increased over the period. Structural factors such as the level of development have been significant in influencing tax revenues. Foreign aid is inversely associated with tax revenues while external debt stock has a significant positive impact on total tax revenues. The trade agreements show some evidence of gaining trade tax revenues. Furthermore, trade openness is found to exhibit a nonlinear effect on trade tax. In addition, a stable macroeconomic environment including exchange rate policies helps strengthen trade tax revenues.

These findings support the view that the lack of tax handles in low-income countries constrains revenue generation and implies that policy aimed at domestic structural shift would help countries in the growth process instead of exclusively depending on external integration policies for structural change and growth. Besides structural features that limit countries from generating revenues from domestic sources, changing consumer demand and market-based production systems are also causing a decline in aggregate income tax. This is confirmed for most Pacific countries in this group as they have seen declining income tax revenue as well as trade tax following trade deregulation. Whether this has happened due to the redistributive influence of trade deregulation or due to tax evasion, remains for further investigation.

The empirical estimation shows that the effect of trade deregulation varies in terms of features of deregulation. While trade deregulation through declining trade tax rates seems to have a significant fiscal cost in Pacific Island countries, regional trade agreements show some support for gaining trade tax revenue. The positive impact of trade deregulation on trade tax appears to be dependent on the level of development as it is observed to be a significant factor in raising trade tax revenues. Thus, countries need to advance regional trade negotiation under the framework of WTO and to produce mutual benefits for the member countries. In addition, simultaneous consideration for comprehensive tax reforms and fiscal consequences of trade policy reforms should be a priority in light of further trade deregulation. Particular attention would be required for this country group which can get hurt from trade tax reduction as trade policy reform given their lack of capacity, including institutional, structural and trade capacities. A mutual and collaborative effort between island nations and the global community including regional trading partners and international financial institutions would be key in aiding these small developing countries to avoid constraints that limit them from taking full benefits of trade deregulation raising government revenue.

## Supporting information

S1 Appendix (DOCX)
