# Supplementary material for: Trade deregulation and fiscal revenue in selected Pacific Island countries
Source: PLoS One. 2025 Jan 2;20(1):e0315733. doi: 10.1371/journal.pone.0315733 (PMC11694958; doi:10.1371/journal.pone.0315733)
Supplement: S1 Appendix — (DOCX) [file pone.0315733.s001.docx]

**Appendix**

**Table A1. Applied tariff rate, weighted mean, all products (%)**

| Year/country | Fiji | Samoa | Solomon Islands | Vanuatu | Papua New Guinea |
| --- | --- | --- | --- | --- | --- |
| 2000 |  |  |  |  | 3.13 |
| 2001 |  |  |  |  | 3.09 |
| 2002 |  |  |  | 20.27 | 2.95 |
| 2003 | 9.51 |  |  | 17.09 | 2.3 |
| 2004 | 10.44 |  |  | 17.14 | 2.1 |
| 2005 |  |  |  | 17.16 | 2.13 |
| 2006 | 8.92 |  |  | 15.81 | 1.89 |
| 2007 | 9.07 |  |  | 18.93 | 1.59 |
| 2008 | 9.97 |  | 14.6 | 18.8 | 2.77 |
| 2009 | 12.15 |  |  | 18.76 | 2.72 |
| 2010 | 11.28 |  |  |  | 2.67 |
| 2011 | 19.88 | 9.85 |  |  |  |
| 2012 | 11.75 | 9.86 |  | 5.53 | 2.57 |
| 2013 | 12.02 | 9.71 |  |  | 2.59 |
| 2014 |  | 9.98 |  |  |  |
| 2015 | 11.12 | 10.28 | 35.65 | 4.84 |  |
| 2016 |  | 10.58 | 14.08 | 7.85 |  |
| 2017 | 16.05 | 10.19 | 18.49 | 8.34 |  |
| 2018 | 16.34 | 10.85 | 30.28 |  | 4.58 |
| 2019 | 23.97 |  | 17.52 |  | 7.75 |
| 2020 | 16.62 | 10.38 | 13.62 | 12.66 | 3.63 |

Source: World Bank’sWorld Development Indicators (2023)

**Table A2. Panel unit root test**

|  | Levin et al. (2002) | | |  | Im et al. (2003) | |
| --- | --- | --- | --- | --- | --- | --- |
| Variable | | Constant | Constant & Time |  | Constant | Constant & Time |
| LTREV | | 2.382 | -1.228 |  | 0.996 | 0.497 |
| LTTAX | | -1.961** | -0.765 |  | -0.706 | 0.278 |
| LGDPC | | -1.456*** | 0.006 |  | -1.277*** | 1.290 |
| LAID | | 1.310 | 2.586 |  | 1.780 | 2.116 |
| LEXD | | -0.116 | -1.321 |  | -0.510 | 0.072 |
| LOPEN | | -0.125 | 1.272 |  | -0.633 | 0.786 |
| LER | | -1.072 | -1.122 |  | 0.419 | -1.059 |
| $\Delta$LTREV | | -3.534* | -7.506* |  | -1.394*** | -7.896** |
| $\Delta$LTTAX | | -2.161** | -1.566** |  | -1.114*** | -6.900** |
| $\Delta$LGDPC | | -2.121** | -2.484* |  | -3.365* | -4.860* |
| $\Delta$LAID | | -2.587** | -1.228* |  | -1.908* | -4.124* |
| $\Delta$LEXD | | -3.068* | -3.538* |  | -3.379** | -6.488** |
| $\Delta$LOPEN | | -5.271* | -6.272* |  | -2.799* | -6.012* |
| $\Delta$LER | -3.465* | | -3.870* |  | -2.850* | -2.162** |

Note: For Levin et al. (2002) test Newey-West bandwidth selection and Bartlett Kernel criteria are used. *, **, and *** denotes 1%. 5%, and 10% statistical significance.

**Table A3. Panel cointegration test**

|  | Equation 2 | | Equation 3 | Equation 4 |
| --- | --- | --- | --- | --- |
| $H_{A}$: common AR coefficients (in-group) | | | | |
| Panel v stats | | -0.367 (0.643) | -.855 (0.196) | -0.241 (0.595) |
| Panel rho stats | | 1.902 (0.971) | 2.681 (0.996) | 1.284 (0.901) |
| Panel PP stats | | -3.109 (0.000)* | -1.674 (0.047)** | -2.858 (0.002)** |
| Panel ADF stats | | -2.207 (0.013)** | -2.638 (0.004)* | -2.392 (0.008)** |
| $H_{A}$: common AR coefficients (inter-group) | | | | |
| Group rho stats | | 2.809 (0.997) | 3.324 (0.999) | 2.371 (0.991) |
| Group PP stats | | -4.111 (0.000)* | -1.825 (0.034)** | -4.781 (0.000)* |
| Group ADF stats | | -4.885 (0.000)* | -2.708 (0.003)** | -6.181 (0.000)* |
| Kao ADF | | -2.407 (0.008)** | -3.522 (0.000)* | -3.411 (0.000)* |

Note: * and ** indicates significance level at 1% and 5%. Probabilities are in parentheses.
